# DropSOAC: Stabilizing Microfluidic Drops for Time-Lapse Quantification of Single-Cell Bacterial Physiology

**DOI:** 10.3389/fmicb.2019.02112

**Published:** 2019-09-24

**Authors:** Shawna L. Pratt, Geoffrey K. Zath, Tatsuya Akiyama, Kerry S. Williamson, Michael J. Franklin, Connie B. Chang

**Affiliations:** ^1^Center for Biofilm Engineering, Montana State University, Bozeman, MT, United States; ^2^Department of Chemical and Biological Engineering, Montana State University, Bozeman, MT, United States; ^3^Department of Microbiology and Immunology, Montana State University, Bozeman, MT, United States

**Keywords:** drop-based microfluidics, single cell, growth rate, lag time, time-lapse imaging, biofilm, heterogeneity, emulsion stability

## Abstract

The physiological heterogeneity of cells within a microbial population imparts resilience to stresses such as antimicrobial treatments and nutrient limitation. This resilience is partially due to a subpopulation of cells that can survive such stresses and regenerate the community. Microfluidic approaches now provide a means to study microbial physiology and bacterial heterogeneity at the single cell level, improving our ability to isolate and examine these subpopulations. Drop-based microfluidics provides a high-throughput approach to study individual cell physiology within bacterial populations. Using this approach, single cells are isolated from the population and encapsulated in growth medium dispersed in oil using a 15 μm diameter drop making microfluidic device. The drops are arranged as a packed monolayer inside a polydimethylsiloxane (PDMS) microfluidic device. Growth of thousands of individual cells in identical microenvironments can then be imaged using confocal laser scanning microscopy (CLSM). A challenge for this approach has been the maintenance of drop stability during extended time-lapse imaging. In particular, the drops do not maintain their volume over time during incubation in PDMS devices, due to fluid transport into the porous PDMS surroundings. Here, we present a strategy for PDMS device preparation that stabilizes drop position and volume within a drop array on a microfluidic chip for over 20 h. The stability of water-in-oil drops is maintained by soaking the device in a reservoir containing both water and oil in thermodynamic equilibrium. This ensures that phase equilibrium of the drop emulsion fluids within the porous PDMS material is maintained during drop incubation and imaging. We demonstrate the utility of this approach, which we label DropSOAC (Drop Stabilization On A Chip), for time-lapse studies of bacterial growth. We characterize growth of *Pseudomonas aeruginosa* and its Δ*hpf* mutant derivative during resuscitation and growth following starvation. We demonstrate that growth rate and lag time heterogeneity of hundreds of individual bacterial cells can be determined starting from single isolated cells. The results show that the DropSOAC capsule provides a high-throughput approach toward studies of microbial physiology at the single cell level, and can be used to characterize physiological differences of cells from within a larger population.

## Introduction

Microbial populations contain cells in a variety of physiological states (Stewart and Franklin, 2008). Even in clonal populations under laboratory conditions, subpopulations of cells may be physiologically different from the rest of the population. This phenomenon is best exemplified by the presence of persister cells, where a minor subset of cells has enhanced resistance to killing by antibiotics, compared to the rest of the cells in the population (Lewis, 2007; Balaban et al., 2013). The persister antibiotic-resistant state is often induced by the activity of self-encoded toxin-antitoxin (TA) systems (Gerdes and Maisonneuve, 2012), where the toxin affects a small percentage of cells, and allows them to tolerate antibiotics that target the greater population. Another example of physiological heterogeneity is the subpopulations of cells that produce colony morphologies that differ from the majority of cells in the community, such as the rugose and mucoid colony morphology variants (Allegrucci and Sauer, 2007; Hansen et al., 2007; McEllistrem et al., 2007). The percentage of persister cells and colony variants increases when bacteria grow in biofilms, or surface-attached communities of bacteria and their extracellular matrices (Stoodley et al., 2002). Reaction and diffusion of nutrients, oxygen, and metabolic products also contribute to physiological heterogeneity of bacteria in biofilm communities (Stewart and Franklin, 2008). These chemicals form gradients within the biofilm and induce microscale variations in gene expression of individual cells. Bacterial physiological heterogeneity poses a significant challenge to the treatment of bacterial infections. In particular, colony variants, persister cells, or dormant cells within biofilms may resist antibiotic treatment or host defenses, and repopulate the communities when treatment is alleviated, resulting in chronic infections (Lyczak et al., 2002).

Technological advances, particularly with regard to miniaturization and single-cell resolution platforms, provide a means to characterize bacterial cell-level heterogeneity. Isolation of single cells for time-lapse observation has been approached through chamber (Leung et al., 2012), flow (Shi et al., 2008; Sun et al., 2011; Dewan et al., 2012), and drop-based microfluidic systems (Huebner et al., 2009; Shim et al., 2009; Khorshidi et al., 2014). Single-cell physiological studies allow direct observation of cell behavior that is otherwise masked by population-level studies (Brehm-Stecher and Johnson, 2004). Therefore, advances in characterizing single-cell physiology complement advances in DNA sequencing technologies that allow single-cell genomics studies. Microfluidic methods, characterized by microscale platforms, minimized reagent volumes, and high-throughput sample preparation, have a distinct advantage for single cell studies, and are suited for research on the physiology of biofilm cell heterogeneity (Weibel et al., 2007; Franklin et al., 2015; Akiyama et al., 2017).

Drop-based microfluidics provides an option that maximizes isolation of single cells and the high-throughput capabilities of microfluidic technology (Chang et al., 2015). Drop-based microfluidics can be used to produce water-in-oil drops with diameters between ten and several hundreds of microns. Single cells in growth medium can be encapsulated in these drops. Drops can be arrayed, held stationary, and imaged using time-lapse microscopy. Previous time-lapse studies involving the imaging of drops to monitor cell growth have used drops larger than 30 μm in diameter (Dewan et al., 2012; Leung et al., 2012; Khorshidi et al., 2014). Larger drops are advantageous in accommodating larger cells, such as algae (Pan et al., 2011) and mammalian cells (Khorshidi et al., 2014). However, smaller drops <20 μm in diameter are preferred to accommodate the growth of single bacterial cells while maximizing the number of drops per field of view. Polydimethylsiloxane (PDMS) is an inexpensive, optically clear polymer commonly used to manufacture microfluidic chips. Although ideal for imaging and rapid fabrication of microfluidic devices (Duffy et al., 1998), PDMS is a porous material that allows the diffusion of fluid from drop emulsions stored in microfluidic devices into the polymer matrix over time (Shim et al., 2007, 2009; Huebner et al., 2009; Schmitz et al., 2009; Pan et al., 2011; Dewan et al., 2012; Amselem et al., 2016). Thus, in time-lapse imaging studies of drops stored in PDMS, the drop volumes do not remain stable due to fluid transport through the porous PDMS matrix. Previous approaches have been developed to mitigate fluid transport through PDMS with varying levels of success. These methods include the incorporation of water reservoirs in PDMS devices (Shim et al., 2007, 2009; Dewan et al., 2012), submersion of devices in water prior to and during use (Pan et al., 2011; Khorshidi et al., 2014), and embedding a diffusion limiting glass layer within the devices above the drop storage areas (Schmitz et al., 2009). However, none of these methods have investigated the stability of a static array of small drops (<20 μm in diameter) for extended time-lapse imaging of single bacterial cells. Such an approach would maximize the number of individual cells that can be imaged and characterized to allow for high-throughput, physiological studies on bacteria.

Here, we present the DropSOAC (Drop Stabilization On A Chip) method for preparing PDMS devices that stabilize <20 μm diameter water-in-oil drops in a static array during time-lapse microscopy for over 20 h. Stability of the position, volume, and geometry of the packed drops relies upon the maintenance of phase equilibrium between the drops and a surrounding fluid reservoir. This phase equilibrium is maintained by soaking a PDMS device in water-saturated oil for at least 24 h before introducing drops into the device. The device is housed in a modified Petri dish with a tight-fitting lid, called a DropSOAC capsule, to prevent evaporation of fluid and maintain phase equilibrium. Filling the capsule with the soaking fluid allows the devices to remain submerged during time-lapse confocal laser scanning microscopy (CLSM) imaging. We demonstrate the utility of this approach for quantifying the heterogeneity of *Pseudomonas aeruginosa* and a *P. aeruginosa* Δ*hpf* mutant derivative with respect to their growth kinetics following resuscitation after starvation. Single cells of *P. aeruginosa* and the Δ*hpf* mutant were encapsulated in drops using a 15 μm drop making device, upon which the drops were introduced into the DropSOAC capsule and incubated at 37°C in a CLSM stage-top incubator. The growth of each fluorescently labeled cell within a drop was quantified and tracked over time using CLSM. Our results demonstrate that drop stability is maintained in the PDMS devices using the DropSOAC approach. We demonstrate that growth rate and lag time heterogeneity can be determined starting from single bacterial cells as inocula, and that our DropSOAC method can capture heterogeneity at the single cell level that might otherwise be masked by bulk measurements of population growth.

## Materials and Methods

### PDMS Microfluidic Device Fabrication

Polydimethylsiloxane (PDMS) microfluidic devices were fabricated using standard soft photolithography processes (Duffy et al., 1998). Photoresist (Microchem SU-8 2015) was patterned onto 76.2 mm silicon wafers (University Wafer) using photomasks (CAD/Art Inc., AutoCAD) to make negatives for PDMS molding. PDMS base and hardener (Dow SYLGARD 184) were mixed at a ratio of 10:1 by mass and poured onto the device molds. The uncured PDMS mix was degassed in a vacuum chamber and cured in a 65°C oven for 1 h. The cured PDMS slabs were removed from the master and ports were punched using a 0.75 mm biopsy punch (EMS Core Electron Microscopy Sciences). The slabs were bonded to 3 in × 2 in glass slides (VWR) for drop-making microfluidic devices and 25 mm × 25 mm type 0 coverslips (VWR) for drop incubation devices. The bonded PDMS and glass devices were baked at 65°C to increase strength of the plasma bonds. After baking, the device channels were filled with hydrophobic treatment (Pittsburgh Glass Works Aquapel), cured for 5 min, and flushed with air. The devices were again baked at 65°C for 1 h to evaporate the remaining hydrophobic treatment.

### DropSOAC Capsule Preparation and Soaking

PDMS and glass drop incubation devices were inserted through 23.5 mm × 23.5 mm laser cut holes (Universal Laser Versa Laser VSL 3.5) in the bottom of 47 mm diameter petri dishes (Millipore Sigma Petri-Pad) and adhered with two-component epoxy (Devcon 5 Minute Epoxy). The resulting DropSOAC capsules were placed unlidded in an airtight container of approximately 400 mL of Novec HFE-7500 fluorocarbon oil (3M, St. Paul MN) and 400 mL of water, and submerged under the denser oil phase. The container was placed in an incubator at 37°C. Unlidded capsules were pre-soaked for a minimum of 24 h in the oil/water bath. After soaking, a DropSOAC capsule was briefly removed from the bath. Drops were injected into the PDMS devices embedded in the capsule from an inverted syringe. Inverting the syringe allows the less dense aqueous drops to float to the syringe outlet. The DropSOAC capsule was returned to the oil/water bath, submerged under the oil phase, and lidded, resulting in a water-saturated oil reservoir around the drop-filled PDMS device. The lidded capsule was removed from the bath, dried with a paper towel, and placed coverslip-side down in an environmental chamber (Pathology Devices Inc., LiveCell) set to 37°C and 85% RH on the stage of an inverted confocal microscope (Leica TSC SP5 II) for imaging. For the studies investigating water only and oil only soaking methods, the DropSOAC capsule was submerged either in 400 mL of oil without water or in 400 mL of water without oil, rather than water and oil in contact with one another. For the no treatment control studies, the DropSOAC capsules were not soaked in fluid prior to or during use and were kept in air.

### Drop Production

Drops were prepared using a 15 μm PDMS flow-focusing microfluidic drop making device. Two different fluids, water as the disperse phase, and HFE7500 (3 M) fluorocarbon oil containing 1.5 wt% 008-FluorSurfactant (RAN Biotechnologies) as the continuous phase, were transported through 0.38 mm ID/1.09 mm OD polyethylene tubing (Scientific Commodities Inc.) from 1 mL syringes (BD Leur-Lok) and 27G × 1/2 needles (BD Precision Glide Needle) to the drop making device. The surfactant used, 008-FluorSurfactant, is composed of a biocompatible PFPE-PEG block copolymer (Holtze et al., 2008). Flow was achieved using a volume-displacement syringe pump (PHD 2000, Harvard Apparatus) at disperse and continuous flow rates of 200 and 600 μL/h, respectively. Drops exited the device through tubing and were collected into an inverted 1 mL syringe (BD Leur-Lok tip) for 15 min.

### Behavior of Water-in-Oil Drops With Various Soaking Methods (No Bacteria)

Water-in-oil drop stability was tested in DropSOAC capsules prepared by soaking in an oil/water bath, soaking in an oil bath, soaking in a water bath, or left untreated, as described in section “DropSOAC Capsule Preparation and Soaking.” Each soaking method was tested in triplicate by filling the prepared devices with water-in-oil drops and imaging over 4.5 h as described in section “Drop Imaging.” During these tests, multiple images of drops held in the DropSOAC capsules (one capsule per trial) were collected every 30 min. For the oil/water soak and the oil only soak, diameter data were randomly sampled from each of the images and compiled across all trials. These images were analyzed using a custom Matlab code to identify drops and determine the drop diameters (data in Supplementary Table S1). The drop diameter data contains values from multiple capsules, devices within the capsules, and positions (fields of view) within the devices. Thus, a mixed effects model was fit to the data set with random effects for the trials (each in a separate capsule) and the positions imaged in each trial to account for the repeated measures from each trial and position, and fixed effects for time and soaking method. Three trials were also tested in the water only soaking method and the no treatment control, but drop diameters could not be quantified due to drop deformation. To quantify drop volumes in DropSOAC capsules prepared by soaking in water, the experiment was repeated in which 500 nM of the fluorescent dye, 5-carboxy-X-rhodamine (ROX), was added to the water drops for CLSM imaging and size quantification using Imaris software.

### Preparation of Drop Phase for Cell Encapsulation

Cells were prepared similarly to the methods described in Akiyama et al. (2017). *P. aeruginosa* PAO1 and its Δ*hpf* derivative that constitutively expresses the green fluorescent protein (GFP) from plasmid (pMF230) (Nivens et al., 2001) were cultured overnight from freezer stock in tryptic soy broth (TSB) amended with 150 μg/mL carbenicillin. Aliquots (120 μL) of overnight cultures were inoculated into 4 ml of TSB without carbenicillin in culture tubes and incubated at 37°C on a roller until the optical density (OD_600_) exceeded 7.0 (CE2041 Spectrophotometer, Cecil Instruments). This OD value corresponds to stationary phase of growth. Aliquots of cultures that resulted in a final concentration of 1.5 × 10^8^ CFU/mL, based on the OD_600_ (approximately 1 mL) were centrifuged, washed twice with phosphate buffered saline (PBS), and resuspended in 25 mL of PBS buffer in a 125 mL baffled flask. Four aliquots of the cell suspension (two of PAO1 and two of Δ*hpf*) were prepared for 0-day starved cell growth studies, or “day zero” studies. Here, 100 μL of cells in PBS were removed from the 125 mL flasks, added to 900 μL of TSB media, and encapsulated into drops. Cells were promptly encapsulated (within 20 min) after reintroduction to media to begin acquiring growth data soon after encapsulation. Two aliquots of cells (one of PAO1 and one of Δ*hpf*) were prepared for 4-day starved cell growth studies, or “day four” studies, in which cells were incubated in PBS at 37°C with shaking at 200 rpm for 4 days. Following incubation, 100 μL of cells in PBS buffer were removed from the 125 mL flasks, added to 900 μL of TSB, and immediately encapsulated into drops. The prepared cell cultures were used as the disperse phase in the drop production method described above.

### *P. aeruginosa* PAO1 and Δ*hpf* Single Cell Growth Studies

As a test case for evaluating drop stability during bacterial growth, and for quantifying bacterial growth curves, we evaluated *P. aeruginosa* PAO1 and Δ*hpf* growth in drops containing TSB growth medium. Diluted cell cultures resuspended in TSB, as described above, were used as the disperse phase in drop production, to achieve approximately one cell per 17 drops. Each DropSOAC capsule holds three arrays, and therefore can be used to evaluate up to three samples simultaneously. In these experiments, two arrays were used, one for PAO1 and one for Δ*hpf*, so that the two strains were assayed simultaneously. Both strains were analyzed prior to starvation, in two separate experiments, and then once after 4 days of starvation in PBS (Akiyama et al., 2017). Three image series were collected for each strain on each array. The change in drop diameters from all time-lapse studies (Supplementary Table S2), which consisted of two experiments of day zero PAO1 and Δ*hpf* and one experiment of day four PAO1 and Δ*hpf*, were analyzed using a mixed effects model, described in section “Behavior of Water-in-Oil Drops with Various Soaking Methods (No Bacteria),” to calculate the average drop size change after 21.5 h. Each field of view using the 20X objective of the CSLM can image approximately 1,150 drops. An additional study with three biological replicates of day four starved Δ*hpf* cell culture (prepared as described in section “Preparation of Drop Phase for Cell Encapsulation”) was conducted to assess percent of cells that resuscitated in drops after 24 h. For these studies, cells were encapsulated in drops, incubated in DropSOAC capsules, and imaged after 24 h. To determine bulk growth rates, PAO1 (pMF230) and PAO1 Δ*hpf* (pMF230) overnight cultures were diluted to an OD_600_ of 0.1 in 200 μl TSB in 96 well plates. Plates were incubated with constant shaking at 37°C for up to 8 h in the SpectraMax190 (Molecular Devices), and OD_600_ was monitored every 10 min. The assay was performed on three biological replicates placed in three independent microtiter plates.

### Drop Imaging

Filled DropSOAC capsules were placed in an environmental chamber (Pathology Devices Inc., LiveCell) at 37°C and 85% RH on an inverted confocal microscope (Leica TSC SP5 II). Drops were imaged with a 20× objective in a 60 μm *z*-stack with 2 μm increments. The middle of the *z*-stack was set to the center of the drops. Images were taken using brightfield and fluorescence imaging every 30 min for the duration of 21.5 or 4.5 h for the bacterial incubation studies and the drop stability studies, respectively. *Z*-stacks were used to capture the entire depth of the drop and to ensure that changes in focal plane from temperature fluctuations were captured. Multiple positions were captured for each study using the “Mark and Find” application within the Leica microscopy software (LAS AF). In the water soaking study with ROX dye, a 63X water immersion objective was used.

### Image Processing and Analysis

The 4D image stacks, composed of three spatial dimensions and time, acquired from confocal imaging of *P. aeruginosa* in drops were processed with the Fiji (Schindelin et al., 2012) distribution of ImageJ (Rueden et al., 2017). A summed *z*-projection of the GFP channel was used to combine the pixel values of all focal planes, capturing fluorescence from bacteria at all locations within the drop. Drop size measurements for each device preparation method were collected with a custom MATLAB script using the brightfield channel. The Fiji plugin TrackMate (Tinevez et al., 2017) was used to identify fluorescent cells in drops and track the change in pixel intensity from the GFP channel, frame by frame, as the cells grew. Pixel intensity is linearly correlated with the concentration of cells within the drops over the range of the PMT output on the confocal microscope. The tracking data collected from TrackMate was then loaded into a custom MATLAB script to plot growth curves for each drop, calculate a maximum growth rate (μ_*max*_), and calculate the lag phase length. Maximum growth rate was calculated from the slope of the linear portion of the growth curve when the natural log of the fluorescence was plotted against time. The lag phase length was determined from the time at the start of image acquisition until the fluorescence within a drop rose above background noise and was tracked by TrackMate. The frequency of cell resuscitation in drop growth assays was determined using TrackMate data. To quantify numbers of cells that resuscitated, the number of drops in which pixel intensity value was low, but non-zero and constant for the duration of incubation was compared to the number of drops in which fluorescence value increased during incubation.

## Results

### The DropSOAC (Drop Stabilization On A Chip) Capsule Design Allows High-Throughput Time-Lapse Imaging of <20 μm Diameter Water-in-Oil Drops

**FIGURE 1:**
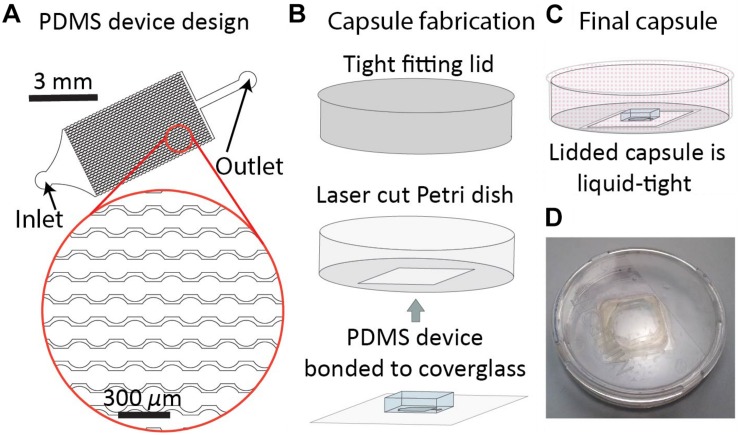
DropSOAC capsule fabrication. **(A)** The drop array device design consists of 30 parallel channels. Design is based on prior work (Schmitz et al., 2009). Each channel connects thirty-one 150 μm diameter wells connected by 75 μm constrictions. Drops are distributed to the channels through a triangular inlet and exit through a shared outlet. **(B)** PDMS devices, plasma bonded to 25 mm × 25 mm type 0 cover glass, are inserted through the base of a 47 mm liquid tight Petri dish and bonded with two component epoxy. **(C)** Lidding the capsule forms a liquid tight seal that allows the PDMS to be submerged under fluid during CLSM while limiting evaporation of the soaking liquid. **(D)** Photograph of the DropSOAC capsule.

The DropSOAC capsule is designed to maximize the number of individual drops observed over time, and keep the PDMS device used to store these drops submerged in liquid. The DropSOAC capsule consists of a PDMS device plasma-bonded to a glass coverslip and interfaced with a modified Petri dish (Figure 1). The PDMS device inside the DropSOAC capsule contains three drop arrays. The channel design in these devices has been used in the past (Köster et al., 2009; Schmitz et al., 2009; Akiyama et al., 2017) and consists of an inlet for drop injection, a feeder channel that distributes the drops to a series of 31 parallel channels, and an outlet channel. Each of the parallel channels consists of a series of 31 circular wells 150 μm in diameter connected by 75 μm constrictions. The parallel channels are offset to maximize the number of channels possible in the device footprint (Figure 1A).

The DropSOAC capsule is a PDMS device/Petri dish hybrid in which a 23.5 mm × 23.5 mm square hole is laser cut into the bottom of a 47 mm × 47 mm Petri dish with a tight fitting lid. The PDMS device, bonded to a 25 mm × 25 mm cover glass, is inserted into the Petri dish through the laser cut hole and adhered with two-component epoxy (Figure 1B). The tight-fitting lid of the Petri dish allows a fluid reservoir to be maintained around the PDMS device after filling the DropSOAC capsule. The filled and lidded DropSOAC capsule prevents spills and the evaporation of reservoir fluid in a format that is compact and well-suited for microscope stage-top incubators (Figures 1C,D).

### Soaking the PDMS Device in an Oil/Water Bath Allows for Drop Stability, Compared to Soaking the Device in No Fluids, Oil Only, or Water Only

**FIGURE 2:**
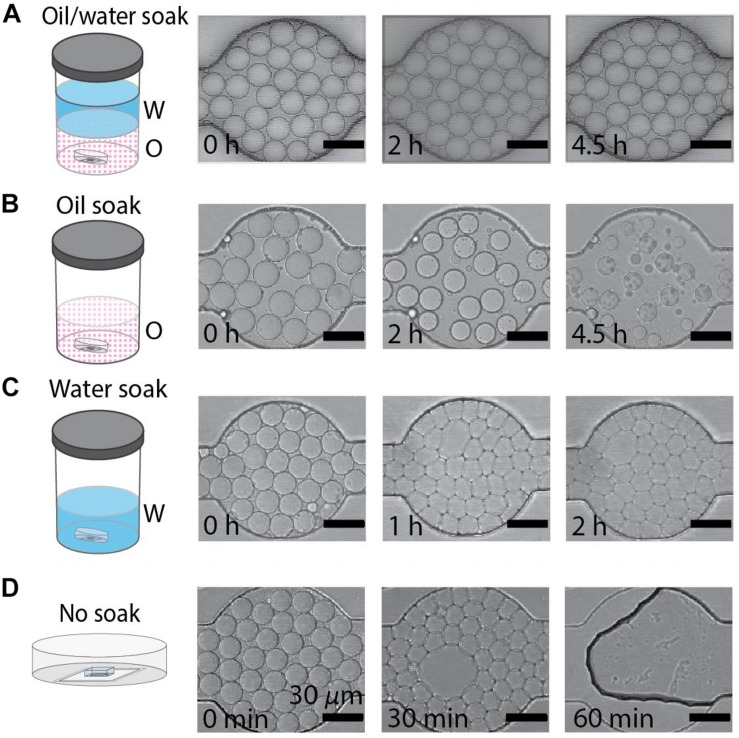
Behavior of water-in-oil drops varies with soaking method. **(A–D)** Water-in-oil drops held in PDMS devices submerged in oil/water, oil, water, or with no soaking treatment. Scale bars are 30 μm. **(A)** Drops maintain diameter and geometry after 4.5 h when the PDMS device is soaked in an oil/water bath. **(B)** Drops shrink over 4.5 h when the PDMS device is soaked in an oil bath. **(C)** The geometry of drops undergoes a rapid transformation from round to faceted polyhedrons when the PDMS is soaked in a water bath. **(D)** Drops destabilize when held in untreated PDMS devices.

The fluid used to soak PDMS devices in the DropSOAC capsule affects the behavior of drops in the PDMS array over extended periods of time. In this study, three soaking solutions were tested: water-saturated oil, oil, and water. An additional study with an unsoaked capsule was conducted to serve as a control for no treatment. The water-saturated oil soak is prepared by making a bath of water and fluorinated oil (HFE7500), approximately 400 mL each, in an air-tight container and allowing the phases to equilibrate until each phase approaches maximum solubility in the other. Since the density of the oil (1.614 g/mL) is higher than that of water, the oil phase sits at the bottom of the bath. Although the oil and water phases are considered immiscible, there is partial solubility of one phase within the other phase. The solubility of the oil phase in water is <4 ppb by weight; the solubility of water phase in oil is 45 ppm by weight (Novec 3M product information). The DropSOAC capsule is soaked in the prepared water-saturated oil for 24 h. During soaking, the water-saturated oil permeates the porous PDMS device. The stability of drops in the DropSOAC capsules was tested by filling the capsules with drops and imaging them over time using CLSM. The DropSOAC capsule soaked in water-saturated oil resulted in stable maintenance of drop size, geometry, and position over 4.5 h of imaging (Figure 2A).

**FIGURE 3:**
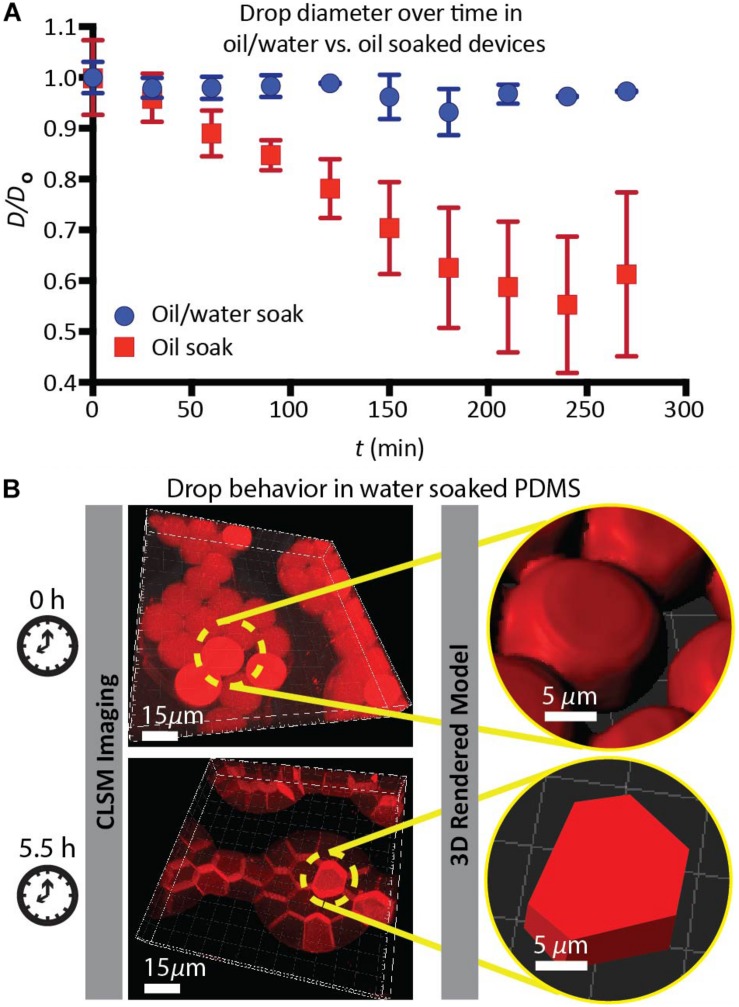
**(A)** Comparison of the average normalized drop diameter *D/D_*o*_* of drops when the PDMS device is soaked in an oil/water bath (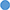, blue dots), versus drops when the PDMS device is soaked in an oil bath (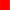, red squares). **(B)** Geometry transformation and volume decrease of drops in the water-soaked PDMS device, imaged by CLSM. Water drops containing ROX fluorescent dye solution is imaged using CLSM and 3D images are used to render models that show shape of the drops at the initial and final time points. Scale bars are 15 μm in CLSM images. Scale bars are 5 μm in the 3D Rendered Model.

By contrast, studies of drops held in DropSOAC capsules that were soaked in oil alone, soaked in water alone, or soaked in no liquid (no treatment control), showed distinct drop destabilization patterns. Drops incubated in an oil prepared DropSOAC capsule shrank over time (Figure 2B). Diameter comparison of drops indicated that oil prepared DropSOAC capsules shrink to 61% ± 16% of their original diameter after 4.5 h (270 min), while the drops stored in water-saturated oil prepared DropSOAC capsules shrink to only 97% ± 1.5% of their original diameter (Figure 3A). The apparent, top-down diameter of drops, *D*, in the device channels is larger than the expected drop diameter of 15 μm due to the buoyancy of the drops causing them to slightly flatten to yield an average initial diameter *D*_0_ = 20.8 μm ± 0.6 μm in the oil/water case and *D*_0_ = 21.0 μm ± 1.5 μm in the oil only case. The final diameters after 4.5 h are *D* = 20.2 μm ± 0.3 μm in the oil/water case and *D* = 12.9 μm ± 3.4 μm in the oil only case.

Drops incubated in a water prepared DropSOAC device showed changes in the shape, undergoing a transition from curved to planar drop interfaces (Figure 2C). The diameter comparison of the water prepared DropSOAC capsules was not possible due to the marked change in drop geometry. These drops transition from slightly flattened spheres at time *t* = 0 min, to drops that are faceted polyhedrons at *t* = 1 h. As the transition was difficult to observe using brightfield microscopy (Figure 2C at *t* = 1 h and *t* = 2 h), in a subsequent study we added an aqueous fluorescent dye (ROX) to the drops to characterize the structural changes of drops, distinguish drop interfaces, and to determine volume changes over time by CLSM. Three-dimensional projections of the fluorescence data show that soaking the DropSOAC capsule in water alone affects drop stability resulting in facetted polyhedrons. The polyhedrons have parallel upper and lower surfaces, and flat sides that are pressed against one another. Image analysis software (Imaris) was used to model the volumes of the drops. Interestingly, the drops in water-soaked PDMS decrease in volume after 5.5 h, to approximately 55% ± 29% (31 initial drops analyzed, 101 final drops analyzed in a single trial) of the original volume (Figure 3B). At the initial time point, CLSM imaging shows flattening of the tops of the drops in water soaked DropSOAC capsules (Figure 3B). Finally, a control study was performed in which drops were held in an untreated DropSOAC capsule. The drops evaporated through the ports and the PDMS, leaving empty channels within the PDMS device in under 1 h (Figure 2D).

### The Water-Saturated Oil DropSOAC Method Allows for Drop Stability in Single-Cell Growth Studies

**FIGURE 4:**
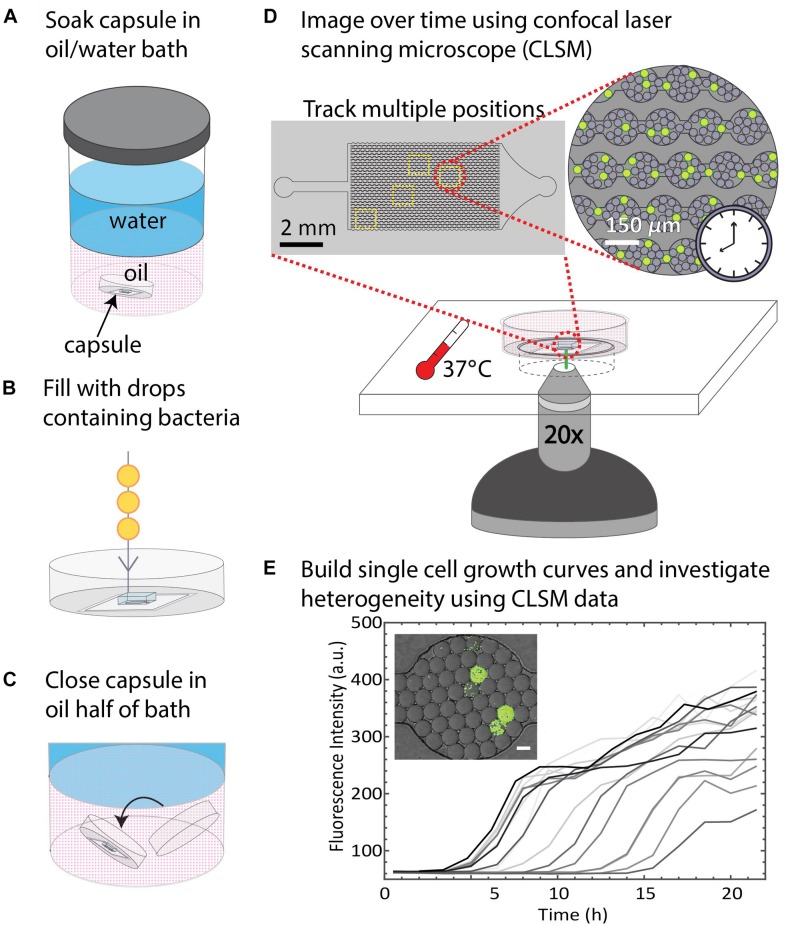
Summary of DropSOAC method and application. **(A)** PDMS-based microfluidic devices embedded in the base of modified Petri dishes (DropSOAC capsules) are soaked in the oil phase of an oil/water bath for 24 h. **(B)** The DropSOAC capsule is removed, and the PDMS device within is filled with drops containing fluorescently labeled bacteria. **(C)** The DropSOAC capsule is lidded under the oil layer of the oil/water soaking bath resulting in a liquid tight capsule filled with water-saturated oil. **(D)** The bacteria-laden drops are imaged using CLSM from within the filled DropSOAC capsule for 21.5 h at 37°C. Multiple positions on the device are imaged, resulting in thousands of drops imaged per experiment. **(E)** Single cell growth curves are constructed from the time-lapse data. These growth curves reveal growth heterogeneity between individual cells.

Since drops remained stable in the DropSOAC capsule soaked in water-saturated oil, we tested the applicability of the DropSOAC approach for studying single cell bacterial growth over time. DropSOAC capsules were soaked under the oil phase of an oil/water bath for 24 h, allowing the porous PDMS device to be filled with water-saturated oil (Figure 4A). After pre-soaking the DropSOAC capsule for 24 h, the capsules were filled with drops that contain single bacterial cells (Figure 4B). Cell loading is governed by Poisson statistics so that there is a low probability that one drop will be inoculated with more than one bacterial cell (Köster et al., 2008). A cell concentration corresponding to a drop loading ratio of one cell to every 17 drops was used in these experiments. After the DropSOAC capsule was filled with drops, it was returned to the oil/water bath and lidded under the oil phase, forming a water-saturated oil reservoir surrounding the PDMS device (Figure 4C). The lidded capsule was removed from the bath and the exterior of the capsule was dried. The lidded capsule was leak-proof and prevented the evaporation of the fluid reservoir around the PDMS. The DropSOAC capsule was then placed in a stage-top incubation chamber on the CLSM and incubated at 37°C. The “Mark and Find” multiple position tool in the confocal microscope software (Leica AF) was used to collect both brightfield and fluorescence images at multiple positions in the PDMS microfluidic array. Using time-lapse programing, each position was imaged every 30 min. The fluorescence signal within the drops was detected as the fluorescently labeled bacteria in the drops grew (Figure 4D). The change in fluorescence signal in each drop was quantified and used as an indicator of cell growth (Figure 4E). Because the growth curves are unique to individual cells within drops, they allow the heterogeneity between cells to be distinguished.

### Growth Heterogeneity of *Pseudomonas aeruginosa* With Single Cells as Inocula

**FIGURE 5:**
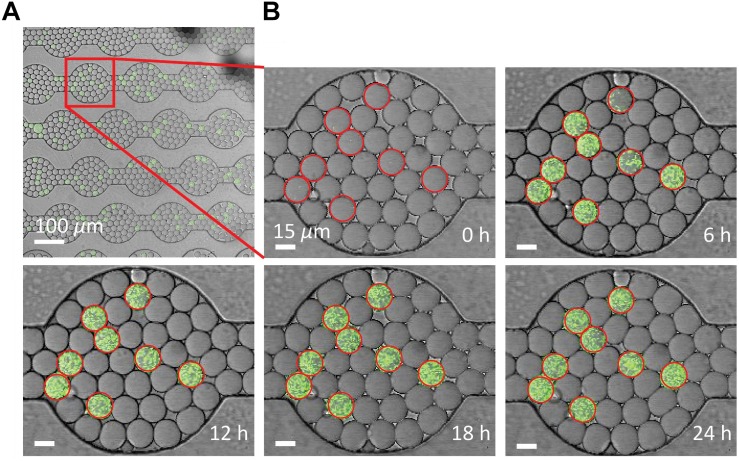
Growth of *P. aeruginosa* PAO1 starved for 0 days in drops over time via CLSM. **(A)** Using a 20X objective lens, each field of view captures approximately 1150 drops. Scale bar is 100 μm. **(B)** *P. aeruginosa* labeled with green fluorescent protein grow from single cells in the drops over 24 h. Growth was quantified from the increase in fluorescent output from each drop. Drops circled in red contain cells. Drop position remains constant over time. Scale bar is 15 μm.

The drop stability of the water-saturated oil DropSOAC capsule allows the study of bacterial growth in drops starting from single cells. Drops containing single bacterial cells remain stable for over 21.5 h, and bacterial growth was observed through an increase in fluorescence over time. To demonstrate this, 0-day starved *P. aeruginosa* PAO1 (pMF230) cells that constitutively express green fluorescent protein (GFP) were washed twice with PBS, resuspended in TBS growth medium, and encapsulated in drops. The drops were injected into the water-saturated oil DropSOAC capsule and imaged every 30 min for 21.5 h by CLSM. Each field of view allowed imaging of approximately 1150 drops (Figure 5A). Increase in fluorescence starting from individual cells was observed over time within stable drops (Figure 5B).

**FIGURE 6:**
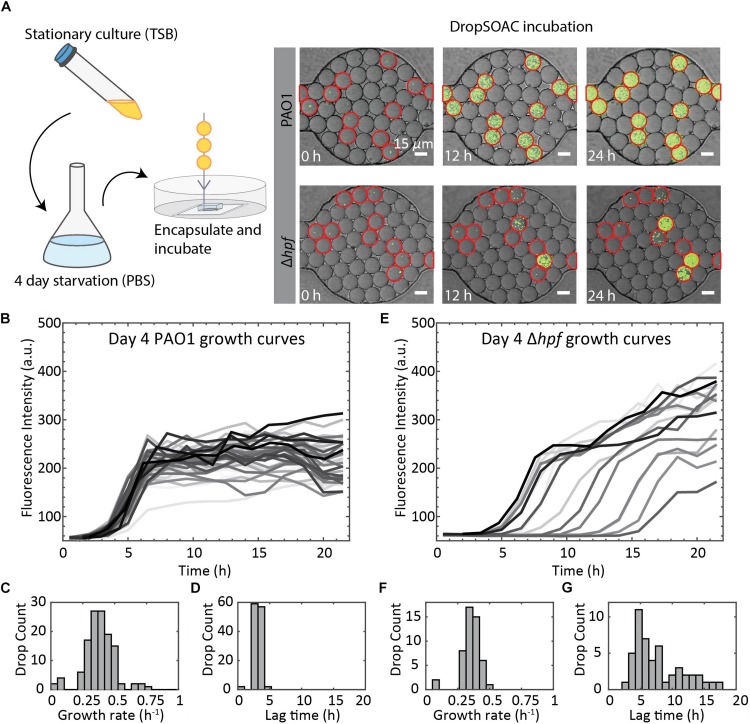
Summary of *P. aeruginosa* PAO1 and Δ*hpf* growth resuscitation studies. **(A)** Cells were starved in PBS for 4 days. After starvation, cells were resuspended in TSB media and encapsulated in drops using a 15 μm drop maker. Drops are incubated in a DropSOAC capsule soaked in water-saturated oil and imaged using CLSM. Growth was monitored as increased fluorescence. Many of the Δ*hpf* cells show delayed growth or no growth following starvation, which can be seen from the image at 12 h. Drops circled in red contain cells. Scale bars are 15 μm. **(B)** Single cell growth curves of *P. aeruginosa* PAO1 following 4 d of starvation. Image shows 80 out of a total of 210 cells analyzed from one field of view, with non-growing cells omitted. **(C)** Histogram showing the distribution of maximum growth rates for *P. aeruginosa* PAO1 following starvation (*N* = 210). **(D)** Histogram showing the distribution of lag phase length of *P. aeruginosa* PAO1 (*N* = 210). **(E)** Single cell growth curves of *P. aeruginosa* Δ*hpf* following 4 d of starvation. Image shows 85 out of a total of 218 cells analyzed from one field of view, with non-growing cells omitted. **(F)** Histogram showing the distribution of maximum growth rates for *P. aeruginosa* Δ*hpf* following starvation (*N* = 218). **(G)** Histogram showing the distribution of lag phase length of *P. aeruginosa* Δ*hpf* (*N* = 218).

To demonstrate the utility of the DropSOAC device for studying growth heterogeneity in *P. aeruginosa* populations, we compare the differences in regrowth of *P. aeruginosa* PAO1 and *P. aeruginosa* (Δ*hpf*), that is deleted for the hibernation promoting factor, following 4 days of starvation (Akiyama et al., 2017). The two strains of *P. aeruginosa* were washed in PBS, as described in the methods, and incubated in PBS for 4 days at 37°C. Day four starved cultures were then encapsulated in drops containing TSB regrowth medium, with approximately one cell in every 17 drops. The drops were injected into the DropSOAC capsule, and the capsule was incubated at 37°C in a stage-top incubator. Drops were imaged every 30 min for 21.5 h using CLSM (Figure 6A). The drops maintained consistent positions and geometry within the device during time-lapse imaging. At time zero, the average drop diameter was 19.6 μm ± 1.4 μm, and after 21.5 h, the average drop diameter was 19.9 μm ± 1.7 μm.

We quantified single-cell growth of bacteria cultured in microfluidic drops using the increase in fluorescence intensities over time for *P. aeruginosa* PAO1 and its Δ*hpf* mutant derivative (Figure 6A). The growth curves were used to calculate the distributions of maximum growth rates, and the length of time required for individual cells to recover from starvation (lag phase). Following 4 days of starvation, *P. aeruginosa* PAO1 regrew in TSB drops with most cells entering exponential phase at approximately 4 h. The drops inoculated with single cells were completely filled with bacteria by 8 h, as indicated by a homogenous, bright fluorescence signal in the entire drop. Regrowth of PAO1 (Figure 6B) had an average growth rate of 0.39 h^–1^ ± 0.11 h^–1^ (Figure 6C) and an average lag phase length of 3.10 h ± 0.62 h (Figure 6D). By contrast, the drops containing the day four starved Δ*hpf* cells had varying levels of fluorescence output at each time point (Figure 6E). Analysis of the Δ*hpf* growth curves yielded an average maximum growth rate of 0.36 h^–1^ ± 0.08 h^–1^ (Figure 6F). By comparison, the growth rate of PAO1 (pMF230) and Δ*hpf* (pMF230) is 0.784 h^–1^ ± 0.042 h^–1^ and 0.689 h^–1^ ± 0.067 h^–1^ when cultured in TSB media in bulk. For the Δ*hpf* cells that were capable of regrowth in drops, the growth rate was similar to that of PAO1. Despite consistent growth rates within the Δ*hpf* population, there was a wide distribution of lag times over the course of the 21.5 h incubation period. The lag times for the Δ*hpf* cells capable of resuscitation ranged from 3 h to 17.5 h (Figure 6E) with an average lag phase length of 7.71 h ± 3.98 h (Figure 6G). Most day four starved Δ*hpf* cells, approximately 86.6% ± 2.5%, did not resuscitate in drops following starvation. Cells that did not resuscitate from starvation were not included in the statistical analyses for growth rate and lag phase length. For each strain, three fields of view were monitored, resulting in approximately 3000 drops and over 200 cells imaged per strain.

## Discussion

### Minimizing Drop Size Maximizes Efficiency of Drop-Based Microfluidic Cell Growth Assays

Previous time-lapse studies involving the imaging of cells in drops have used drops larger than 30 μm in diameter (Dewan et al., 2012; Leung et al., 2012; Khorshidi et al., 2014). Smaller drops, <20 μm in diameter, are preferred to accommodate the growth of single microbial cells since they maximize the number of drops per field of view within the array. In our study, we observe approximately 1150 drops per field of view using a 20X objective lens. We monitored three fields of view per sample in this study, however, up to 42 distinct fields of view in each array are available for imaging. This corresponds to approximately 48,300 drops that can be imaged in one array. Each DropSOAC capsule can hold up to three arrays, therefore, each capsule allows for the imaging a total of approximately 145,000 drops. Theoretically, cell concentrations that correspond to one cell per drop for 50 μm drop diameters and 15 μm drop diameters are 1.5 × 10^7^ cells/mL and 5.7 × 10^8^ cells/mL, respectively. Reducing the drop diameter from 50 μm to 15 μm allows an order of magnitude more cells to be studied in the same volume for single cell drop-based assays, which is important when investigating rare single cells in a population.

The device used in this work is a modification of a previously designed drop maker and array, which was designed to hold individual drops in wells connected by constrictions (Schmitz et al., 2009). Here, the drop maker is independent of the DropSOAC device. Separating the devices allows us to incubate 15 μm drops in 150 μm diameter wells with 75 μm width constrictions (Figure 1) and allows multiple bacterial strains to be studied simultaneously as one DropSOAC device contains three arrays. This system is intended to hold many drops in the wells and channels to increase the number of drops imaged per field of view. In addition, the resolution limitation of soft photolithography depends on the minimum mask resolution. Our mask printing source limits features to approximately 10 μm at their highest resolution, thus making it difficult to accommodate single drops with diameters <20 μm in features that allow for one drop per well. These same limitations make the fabrication of other drop arrays, such as floating arrays (Shim et al., 2009; Khorshidi et al., 2014; Håti et al., 2016), anchors (Abbyad et al., 2011), and hydrodynamic drop traps (Shi et al., 2008; Bai et al., 2010; Bithi and Vanapalli, 2010; Sun et al., 2011), unrealistic for small scale microfluidic drops.

### PDMS Devices Require Oil/Water Soaking Treatment to Successfully Incubate Drops

The diffusion of water in stored emulsions within PDMS devices is a widely observed challenge for maintaining drop volumes (Shim et al., 2007, 2009; Huebner et al., 2009; Schmitz et al., 2009; Pan et al., 2011; Dewan et al., 2012; Amselem et al., 2016), which is also demonstrated in this work (Figures 2B–D). In prior work (Shim et al., 2007, 2009; Pan et al., 2011; Dewan et al., 2012; Khorshidi et al., 2014), water only reservoirs and soaking techniques have been used to maintain drop volumes. Similarly, we submerge the DropSOAC device both prior to and during imaging, but unlike prior work, water-saturated oil instead of water is used as the submersion liquid. The use of water-saturated fluorocarbon oil as the submersion liquid requires that the device also be designed to limit the evaporation of the highly volatile oil, which can be accomplished using a tight-lidded Petri dish in the DropSOAC capsule. Lidding the device produces a liquid-tight, evaporation-reducing chamber that can be filled with fluid to maintain PDMS device submersion during imaging. The DropSOAC capsule provides a simple solution that allows for compatibility with CLSM and microscope stage-top incubation. The oil/water soak in the DropSOAC method resulted in no significant change in drop volume after 21.5 h of storage, which was demonstrated to be more effective than water only soaking methods used in previous studies of drops in a similar fluorinated oil continuous phase (3 M Fluorinert FC-40). These studies exhibited up to a 12% decrease in volume over 16 h (Amselem et al., 2016), and a 10% decrease in volume over 20 h (Shim et al., 2009). In addition, the DropSOAC method prevents the need for more complicated device fabrication, such as embedding a diffusion limiting glass layer above the drop storage areas (Schmitz et al., 2009), or a custom-made glass chamber (Boitard et al., 2012).

### The DropSOAC Method for Soaking PDMS Results in Better Drop Stability Than in Oil or Water Alone

We observe varying drop stability depending on the fluid used to soak and fill the DropSOAC capsule. This is due to the partial miscibility of the water phase in the fluorocarbon oil phase and vice versa. The stability of the drop emulsion depends upon whether the drops are in contact with (i) water-saturated oil, (ii) oil only, or (iii) water only reservoirs in the surrounding PDMS matrix. (i) Drops incubated in PDMS soaked in a water-saturated oil bath maintain their drop volume, position, and geometry during 4.5 h of stage top incubation and CLSM imaging (Figure 2A). Furthermore, when applied to single cell studies of *P. aeruginosa* in drops, drops remained stable for 21.5 h. In the oil/water system, the PDMS is filled with water-saturated oil by placing the DropSOAC device in a large reservoir containing oil in contact with water (approximately 400 mL of each fluid) for at least 24 h at 37°C. This ensures phase equilibrium in which the concentration of each phase in the other phase approaches maximum solubility. Drops stored in a device that is soaked with oil/water solution maintain their volume because the thermodynamically driven, spontaneous transport of water from drops into the surrounding oil is minimized with an already water-saturated oil phase. (ii) By contrast, drops incubated in PDMS soaked in unsaturated oil shrink over 4.5 h while maintaining spherical geometry (Figure 2B). If given more time, the drops shrink until they are no longer observable. This is a result of water diffusion into the oil phase to achieve phase equilibrium in the system. Further experimentation is necessary to measure the solubility of the water in the fluorocarbon phase and to determine the kinetics of this transport. (iii) Finally, drops incubated in PDMS soaked in water also shrink and undergo geometric changes from flattened spheres to polyhedral volumes with facetted faces in which the drops are compressed against one another. This transition occurs rapidly, within the first 30 min after the drops are introduced into the device (Figure 2C shows *t* = 1 h, but the onset of this transition happens earlier). We hypothesize that drop shrinkage and compression are due to transport of both water and oil from the emulsion to the water reservoir in the PDMS. Drop shrinkage may occur due to coarsening of the small water drops into the larger water reservoir in the PDMS, while drop compression may occur due to surfactant depletion forces or drainage of the oil phase between neighboring drops, as observed in a similar system (Courtois et al., 2008). In the surfactant depletion mechanism, the oil phase of the drop emulsion solubilizes into the surrounding PDMS water reservoir, concentrating the surfactant in the oil phase. The increased surfactant concentration promotes the formation of reverse micelles. The presence of reverse micelles in the oil phase is expected, as our surfactant concentration is at 1.5 wt%, which is greater than the critical micelle concentration (CMC) of the surfactant (Wagner et al., 2016; Scanga et al., 2018). Thus, increased surfactant concentration should create additional micelles in solution. These micelles are capable of inducing depletion-attraction aggregation of drops due to the localized exclusion of micelles between drop interfaces. The micelles may also aid in transport of water from the drops to the surrounding PDMS water reservoir. Further experimentation is necessary to test the presence and effect of micelles; however, similar observations have been made in which drops aggregate due to absorption of the oil phase into the PDMS (Courtois et al., 2008).

### The DropSOAC Method Allows for Analysis of Single Cell Growth Kinetics and Quantification of Heterogeneity in Microbial Populations

Using the DropSOAC device to stabilize drops, we were able to monitor growth heterogeneity within clonal populations of *P. aeruginosa* PAO1 and its Δ*hpf* mutant derivative. Our results show that PAO1 has a small standard deviation for the lag time, and that growth curves were tightly clustered. We are also able to track cells that did not grow. Though the average growth rate of both the wild-type and the Δ*hpf* mutant cells were similar under these conditions (0.38 h^–1^ ± 0.12 h^–1^ and 0.36 h^–1^ ± 0.08 h^–1^), the lag time following 4 days of starvation differed. Consistent with our previous study (Akiyama et al., 2017), 86.6% ± 2.5% of the Δ*hpf* mutant cells were unable to recover from starvation and remained as single cells. The cells that recovered had extended and variable lag times, ranging from 3 h to 17.5 h. The Δ*hpf* strain lacks the hibernation promotion factor protein, a ribosomal accessory protein that helps maintain ribosome integrity during starvation. The heterogeneity in lag time in the Δ*hpf* mutant indicates that different cells have varied ability to perform *de novo* protein synthesis and grow following starvation (Akiyama et al., 2017).

### The DropSOAC (Drop Stabilization on a Chip) Approach Is a Simple Method Used to Stabilize Thousands of 15 μm Diameter Drops on a Chip

The DropSOAC approach allows for real-time data acquisition of drops in a static array, circumventing the need to sample drops at various time points. We have demonstrated that DropSOAC allows observation of up to hundreds of single cells in <20 μm diameter drops to quantify heterogeneity in recovery rate of starved bacterial cells. Approximately 145,000 drops of this size can be observed using one DropSOAC capsule. Larger designs can potentially accommodate up to millions of drops as the methods of soaking the PDMS in phase-equilibrated solutions are applicable to larger devices. The advantage of this method is in its simplicity. Compared to other microfluidic fabrication methods used to store drops on chip, such as glass fabrication (Boitard et al., 2012), pumps and valves to compartmentalize cells (Thorsen et al., 2002; Leung et al., 2012), multilayer PDMS devices with water reservoirs (Dewan et al., 2012; Leung et al., 2012), floating or sunken arrays (Shim et al., 2009; Khorshidi et al., 2014; Håti et al., 2016), or fabricating channels on a microfluidic device to trap single cells (Balaban et al., 2004; Rowat et al., 2009; Wang et al., 2010), the DropSOAC approach simply uses a tight-lidded Petri dish to maintain fluid phase equilibrium in the PDMS surrounding the drops. The enclosed, transportable, and compact DropSOAC capsule allows drops to remain stationary and maintain their size over long hours of time-lapse imaging. Greater than one order of magnitude more drops can be stored on chip using DropSOAC compared to prior work (Shi et al., 2008; Huebner et al., 2009; Schmitz et al., 2009; Shim et al., 2009; Bithi and Vanapalli, 2010; Sun et al., 2011; Dewan et al., 2012; Leung et al., 2012; Khorshidi et al., 2014) This is due to the smaller size of these drops and the ability to pack them together so that they are neighboring, while simultaneously stabilizing the drops against coalescence, coarsening, shrinking and aggregation. We have not tested the stability of the drops on chip for over 21.5 h, as this time span was long enough to capture the growth dynamics of the bacterial cells. However, we hypothesize that drops will remain stable as long as the DropSOAC capsule is completely sealed to evaporation and the contents of the drops do not affect drop stability. In the latter case, examples could include bacteria producing molecules that change the surface tension between drops, and osmotic pressure differences between drops containing bacteria and drops containing media leading to coarsening of the drops.

Drop stability during storage in PDMS devices is a challenge observed for water-in-oil emulsions, regardless of the oils used, such as other fluorinated oils (3 M Fluorinert) (Schmitz et al., 2009; Shim et al., 2009, 2011; Knowles et al., 2011; Dammann et al., 2012; Lagus and Edd, 2013; Khorshidi et al., 2014; Shemesh et al., 2014) or mineral oil (Courtois et al., 2008; Courtois et al., 2009; Huebner et al., 2009; Bai et al., 2010; Dewan et al., 2012). Applying the DropSOAC approach used here to other systems may provide improved emulsion stability. In addition, other emulsion formulations may provide even better drop stability than demonstrated in our system, such as emulsions formulated with a less volatile oil as the continuous phase, or oil and water phases in which the partial solubility of one phase in another is much lower. Certainly, other device materials such as glass (Boitard et al., 2012) and optical adhesive (Abbyad et al., 2011) can be used to prevent evaporation; however, these materials may have other disadvantages in their lack of optical transparency, resolution, ease of fabrication, and oxygen permeability. Further investigation is necessary to test the effects of laser exposure on the cells in drops when imaging under confocal microscopy, and to test the effects of oxygen and nutrient limitation in the DropSOAC capsule. Nonetheless, in this work, we have demonstrated the utility of DropSOAC in stabilizing tens of thousands of drops on chip for long-term, time-lapse imaging over 20 h. We used DropSOAC to obtain growth curves of hundreds of single cells, providing insight into the behavior of individual cells within a clonal population, which can easily be scaled up to thousands of cells. Other advantages of the DropSOAC approach include the ability to recover the drops from the device for downstream analysis or sequencing and the general applicability of the soaking method, which can be easily adapted to other types of geometries and drop sizes on chip.

## Conclusion

The DropSOAC (Drop Stabilization On A Chip) method for preparing PDMS microfluidic devices stabilizes the position, volume, and geometry of <20 μm diameter water-in-oil drops in a static array for over 20 h. This is achieved by soaking the PDMS device in a water-saturated oil phase using a simple modified Petri dish with a tight-fitting lid. The DropSOAC method maintains phase equilibrium of the drop emulsion within the porous PDMS material structure throughout the course of drop storage and imaging. With this approach, the heterogeneity in growth rate and lag time of hundreds of cells within a clonal population of bacteria and its mutant derivative can be quantified using time-lapse confocal imaging. We expect that the DropSOAC approach can be widely applied toward the incubation and imaging of other microorganisms to observe individual cell growth kinetics of a population over time.

## Data Availability

All datasets generated for this study are included in the manuscript/Supplementary Files.

## Author Contributions

SP, TA, KW, MF, and CC designed the study. SP, TA, and KW conducted the laboratory experiments. GZ and SP analyzed the data. SP, MF, and CC wrote the manuscript with contributions from all other authors.

## Conflict of Interest Statement

The authors declare that the research was conducted in the absence of any commercial or financial relationships that could be construed as a potential conflict of interest.
